# Rhizospheric and Endophytic Plant Growth-Promoting Bacteria Associated with *Coffea arabica* L. and *Coffea canephora* Pierre ex Froehner: A Review of Their Agronomic Potential

**DOI:** 10.3390/microorganisms13112567

**Published:** 2025-11-11

**Authors:** Marisol Ramírez-López, Angélica Bautista-Cruz, Arcelia Toledo-López, Teodulfo Aquino-Bolaños

**Affiliations:** 1 Doctoral Programme in Conservation and Use of Natural Resources, Instituto Politécnico Nacional, CIIDIR-Oaxaca, Hornos 1003, Santa Cruz Xoxocotlán 71230, Oaxaca, Mexico; mramirezl1700@alumno.ipn.mx; 2 Instituto Politécnico Nacional, CIIDIR-Oaxaca, Hornos 1003, Santa Cruz Xoxocotlán 71230, Oaxaca, Mexico; artoledol@ipn.mx (A.T.-L.); taquino@ipn.mx (T.A.-B.)

**Keywords:** *Bacillus*, biocontrol, biofertilization, endophytic bacteria, microbial consortia, *Pseudomonas*, rhizobacteria

## Abstract

Plant growth-promoting bacteria (PGPB) associated with *Coffea arabica* L. and *Coffea canephora* Pierre ex Froehner offer a viable strategy to reduce synthetic inputs and enhance resilience in coffee agroecosystems. This review synthesizes evidence from the past decade on rhizosphere-associated and endophytic taxa, their plant growth-promotion and biocontrol mechanisms and the resulting agronomic outcomes. A compartment-specific core microbiome is reported, in the rhizosphere of both hosts, in which *Bacillus* and *Pseudomonas* consistently dominate. Within endophytic communities, *Bacillus* predominates across tissues (roots, leaves and seeds), whereas accompanying genera are host- and tissue-specific. In *C. arabica*, endophytes frequently include *Pseudomonas* in roots and leaves. In *C. canephora*, root endophytes recurrently include *Burkholderia*, *Kitasatospora* and *Rahnella*, while seed endophytes are enriched for *Curtobacterium*. Functionally, coffee-associated PGPB solubilize phosphate; fix atmospheric nitrogen via biological nitrogen fixation; produce auxins; synthesize siderophores; and express 1-aminocyclopropane-1-carboxylate deaminase. Indirect benefits include the production of antifungal and nematicidal metabolites, secretion of hydrolytic enzymes and elicitation of induced systemic resistance. Under greenhouse conditions, inoculation with PGPB commonly improves germination, shoot and root biomass, nutrient uptake and tolerance to drought or nutrient limitation. Notable biocontrol activity against fungal phytopathogens and plant-parasitic nematodes has also been documented. Key priorities for translation to practice should include (i) multi-site, multi-season field trials to quantify performance, persistence and economic returns; (ii) strain-resolved omics to link taxa to functions expressed within the plant host; (iii) improved bioformulations compatible with farm management and (iv) rationally designed consortia aligned with production goals and biosafety frameworks.

## 1. Introduction

Globally, coffee cultivation is one of the most significant economic activities and is regarded as the second most traded commodity after oil [1,2,3]. Among the 125 species within the *Coffea* genus, only 2 hold major global commercial value: *Coffea arabica* L. (arabica coffee) and *Coffea canephora* Pierre ex Froehner (robusta coffee), which account for approximately 64 and 35% of global production, respectively [1,4,5]. In recent decades, the expansion of conventional coffee farming has led to a reduction in shaded coffee systems, with potential negative impacts on biodiversity and ecosystem services. Furthermore, the intensification of shaded coffee production is typically associated with increased use of synthetic agrochemicals, including fertilizers, pesticides and herbicides [6]. In particular, the excessive application of synthetic fertilizers in agricultural systems has degraded soil quality and contributed to freshwater contamination due to phosphorus (P) and nitrogen (N) runoff [7,8]. A sustainable alternative to synthetic inputs involves the use of biological agents such as plant growth-promoting bacteria (PGPB). Reported PGPB genera include *Pseudomonas*, *Burkholderia*, *Azospirillum*, *Arthrobacter*, *Azotobacter*, *Klebsiella*, *Serratia*, *Alcaligenes*, *Bacillus* and *Enterobacter* [9]. PGPB constitute a diverse group of bacteria capable of enhancing plant growth and increasing tolerance to both biotic and abiotic stresses when associated with plants [10,11]. Based on their colonization niches, these bacteria are categorized as rhizospheric (residing around the roots), endophytic (inhabiting internal plant tissues), carpospheric (associated with fruit surfaces and interiors) and epiphytic (living on external plant surfaces such as leaves, stems and flowers) [12,13]. PGPB promote plant growth through both direct and indirect mechanisms. Direct mechanisms include enhanced nutrient acquisition via solubilization (e.g., P, potassium, iron), biological nitrogen fixation (BNF), phytohormone synthesis [auxins, particularly indole-3-acetic acid (IAA), as well as cytokinins and gibberellins], siderophore production and 1-aminocyclopropane-1-carboxylate (ACC) deaminase activity. Indirect mechanisms confer protection against phytopathogens and improve stress tolerance through the production of antibiotics and lytic enzymes, modulation of ethylene levels and activation of induced systemic resistance (ISR) in plants [14].

Despite growing insights into the roles of PGPB across crop species, evidence specifically addressing their application in coffee remains comparatively fragmented. This review synthesizes evidence from the past decade on rhizosphere-associated and endophytic taxa in *C. arabica* and *C. canephora*, their plant growth-promotion and biocontrol mechanisms and the resulting agronomic outcomes. Searches were performed in Scopus, Web of Science Core Collection (Clarivate), ScienceDirect, Google Scholar and PubMed. Search strings combined controlled and free-text terms and synonyms using Boolean operators; for example, (*Coffea* OR coffee OR “*Coffea arabica*” OR “*Coffea canephora*”) AND (“plant growth-promoting bacteria” OR PGPB OR PGPR OR rhizobacteria OR endophyte OR endophytic) AND (“biological nitrogen fixation” OR “phosphate solubilization” OR siderophore OR “indole-3-acetic acid” OR IAA OR “ACC deaminase” OR biocontrol OR “induced systemic resistance”). Evidence eligible for inclusion comprised observational and experimental studies conducted in vitro, in nurseries, in greenhouses and in the field, with publications available in English, Spanish or Portuguese. After deduplication, titles, abstracts and full texts were screened. Records were retained if they met (i) taxonomic identification at the genus, species, or strain level and at least one of the following: (ii) quantitative evidence of plant growth-promotion traits (e.g., IAA production, phosphate solubilization, siderophore production, biological nitrogen fixation, ACC deaminase activity); or (iii) demonstrated plant-level effects under nursery, greenhouse or field conditions (growth, nutrient uptake, yield, stress tolerance) with appropriate controls. Conference abstracts, non-peer-reviewed preprints and studies lacking clear methodology or verifiable quantitative metrics were excluded.

## 2. Mechanisms of Action of Plant Growth-Promoting Bacteria

PGPB interact beneficially with their host plants through a range of biochemical and physiological mechanisms that facilitate plant growth. These interactions are typically categorized into two types: direct and indirect mechanisms. Direct mechanisms enhance nutrient acquisition and stimulate phytohormone synthesis, whereas indirect mechanisms are associated with protection against phytopathogens and abiotic stress [15,16].

### 2.1. Direct Mechanisms of Action of Plant Growth-Promoting Bacteria

PGPB can synthesize a broad spectrum of phytohormones, notably auxins, gibberellins and cytokinins [17]. Among auxins, IAA plays a central role in regulating key physiological processes, including cell elongation and division, vascular tissue differentiation, apical dominance, root system development and the gravitropic response, that is, the plant’s ability to perceive and respond to gravity by adjusting the orientation of organ growth. PGPB synthesize IAA from the amino acid tryptophan through various biosynthetic pathways, including the indole-3-pyruvic acid and indoleacetamide pathways [18]. In coffee, inoculation with *Azospirillum brasilense* has been shown to enhance root growth and seedling biomass, effects commonly attributed to the production of IAA and gibberellins, as well as to associative BNF [19].

Cytokinins regulate diverse developmental processes, including apical dominance, cell proliferation, root elongation, seed germination, xylem and chloroplast differentiation, reproductive development, leaf senescence and plant–pathogen interactions [20]. In coffee, epiphytic and endophytic microbes, particularly *Methylobacterium* spp. are established cytokinin producers, consistent with a hormone-mediated route to growth promotion [21].

Gibberellins, on the other hand, promote shoot elongation and play a crucial role in regulating seed germination [22]. Another important feature of many PGPB is their ability to produce ACC deaminase, an enzyme that mitigates the growth-inhibitory effects of ethylene [23]. Under stressful conditions, plants tend to accumulate ACC, the immediate precursor of ethylene. This accumulation leads to increased ethylene production, which can trigger physiological responses such as senescence, chlorosis, leaf abscission and inhibition of shoot and root growth. PGPB that produce ACC deaminase degrade ACC into ammonia and α-ketobutyrate, thereby reducing ethylene synthesis and alleviating its negative effects on plant development [16,24]. Recent work demonstrates that rhizosphere strains from *C. arabica* producing ACC deaminase improve drought tolerance, emphasizing the relevance of this pathway under water-deficit conditions [25].

BNF is another key direct mechanism by which PGPB enhance plant development [26]. Species of *Rhizobium* establish symbiotic relationships with plants from the orders Fabales, Fagales, Cucurbitales and Rosales, fixing atmospheric nitrogen (N_2_) within root nodules and converting it into ammonium (NH_4_^+^), a form readily assimilated by plants [27,28]. In addition to these symbiotic associations, free-living and endophytic nitrogen-fixing bacteria, such as *Azospirillum* and *Azotobacter*, also contribute to nitrogen fixation, albeit generally with lower efficiency than their symbiotic counterparts [22].

Phosphate solubilization is another essential mechanism through which PGPB enhance plant development, particularly because P is often present in insoluble forms that limit its availability to plants. PGPB improve P uptake by solubilizing inorganic phosphate compounds and mineralizing organic P sources [29]. Genera such as *Bacillus*, *Pseudomonas*, *Rhizobium* and *Enterobacter* are known for their ability to convert insoluble P into plant-assimilable forms, including monobasic (H_2_PO_4_^−^) and dibasic (HPO_4_^2−^) phosphate ions [30,31]. Organic acids, including citric acid, gluconic acid and 2-ketogluconic acid, play a crucial role in this solubilization process. In alkaline soils, these acids lower the pH of the rhizosphere, thereby dissolving otherwise unavailable phosphates. In acidic soils, they act as chelating agents, binding iron (Fe^3+^) and aluminum (Al^3+^) ions to prevent the formation of insoluble phosphate complexes, ultimately enhancing P bioavailability. Additionally, PGPB produce hydrolytic enzymes such as phosphatases and phytases, which mineralize organic P compounds into inorganic forms that plants can readily absorb [32,33].

Iron acquisition by plants depends on the formation of ferric iron–siderophore complexes, which enhance the solubilization and uptake of this otherwise poorly soluble nutrient. Siderophores are low-molecular-weight chelating compounds synthesized by bacteria, fungi and certain plants to capture and transport iron in environments where it is limited or exists in insoluble forms [34,35]. Siderophores produced by PGPB increase iron bioavailability by binding to insoluble ferric iron (Fe^3+^) in the soil, forming soluble complexes that can be taken up by both plants and bacteria through specific membrane receptors [36]. In bacteria, these ferric iron-siderophore complexes are recognized by outer membrane proteins and transported into the cell via energy-dependent systems such as the TonB complex and ATP-binding cassette (ABC) transporters. Once internalized, the iron is released and reduced to its ferrous form (Fe^2+^) within the periplasm or cytoplasm, depending on the organism’s specific assimilation pathway [37]. This mechanism of iron acquisition not only enhances plant nutrition but also provides PGPB with a competitive advantage for colonization and persistence in the rhizosphere, thereby supporting improved plant growth in iron-deficient soils. Fluorescent *Pseudomonas* spp., commonly detected in coffee tissues, are well-known producers of high-affinity siderophores, most notably pyoverdine, that promote iron acquisition when bioavailable iron is scarce [5].

### 2.2. Indirect Mechanisms of Action of Plant Growth-Promoting Bacteria

PGPB employ several indirect mechanisms to protect plants from phytopathogens and to strengthen their resistance responses. One of the central features of their biocontrol activity is the synthesis of antimicrobial compounds that allow them to inhibit pathogen growth in the rhizosphere. Bacterial genera such as *Bacillus* and *Pseudomonas* are especially well-known for producing these substances, which include lipopeptides, exoenzymes and volatile organic compounds with strong antifungal and antibacterial properties [38].

In addition, PGPB secrete hydrolytic enzymes that degrade structural components of pathogenic fungal and bacterial cell walls [39]. The most relevant enzymes include chitinases, pectinases, cellulases, glucanases and proteases. Chitinases hydrolyze chitin, a key structural polysaccharide in fungal cell walls; β-1,3-glucanases target β-glucans, further destabilizing the fungal wall and leading to lysis and proteases degrade structural proteins that contribute to the stability and integrity of pathogen cell envelopes [31,40]. Numerous bacterial taxa including the genera *Bacillus*, *Brachybacterium*, *Burkholderia*, *Caballeronia*, *Cellulomonas*, *Chromobacterium*, *Curtobacterium*, *Enterobacter*, *Herbaspirillum*, *Kitasatospora*, *Lechevalieria*, *Leifsonia*, *Luteibacter*, *Microbacterium*, *Micrococcus*, *Mycobacterium*, *Sphingomonas*, *Staphylococcus*, *Bradyrhizobium*, *Mycolicibacterium*, *Nocardia*, *Sphingobium*, *Pseudomonas*, *Rhizobium*, *Kocuria*, *Methylobacterium*, *Sinomonas*, *Paenibacillus*, *Nakamurella*, *Streptomyces* and *Paracoccus* have been reported to produce hydrolytic enzymes such as chitinases, gelatinases, lipases and proteases [41,42].

Another biologically important compound produced by some PGPB is hydrogen cyanide (HCN), a volatile, toxic secondary metabolite synthesized from glycine in a reaction catalyzed by HCN synthase. HCN disrupts cellular respiration in pathogens by inhibiting key enzymes of the electron transport chain, ultimately impeding oxygen utilization and leading to pathogen inhibition or death [43]. HCN production by *Pseudomonas* spp. and other rhizobacteria associated with coffee has been documented in *C. arabica* from Ethiopian forests, together with strong siderophore production that restricts pathogen growth under iron limitation [44].

PGPB also enhance plant immunity through a process known as ISR, a defense response that primes the plant’s immune system in the absence of a current pathogen attack. This mechanism is primarily mediated by the jasmonic acid and ethylene signaling pathways [45]. Furthermore, PGPB can interfere with quorum sensing (QS), a cell-density-dependent communication system used by many pathogenic bacteria to regulate virulence gene expression and biofilm development. PGPB disrupt QS through a process called quorum quenching, which involves degrading or inactivating signaling molecules such as N-acyl-homoserine lactones (AHLs), thereby preventing pathogens from synchronizing their harmful activities [38]. Several lines of evidence support the role for ISR in coffee. Reviews on coffee leaf rust management identify *Bacillus*-derived lipopeptides, particularly surfactins and fengycins, as elicitors of ISR. Consistent with this, applications of *Bacillus subtilis* and *Pseudomonas putida* have been shown to reduce *Hemileia vastatrix* infection in bioassays [46].

Indirect mechanisms highlight the central role of PGPB in phytoprotection. Yet the host plant also shapes plant-microbe interactions. In coffee, the metabolic composition of *Coffea* tissues is a key determinant of these associations. Secondary metabolites characteristic of *C. arabica* and *C. canephora* including caffeine, chlorogenic acids, phenolic acids (caffeic, ferulic and p-coumaric) and flavonoids such as quercetin and kaempferol exhibit antimicrobial and antioxidant activities that modulate microbial behavior in the rhizosphere and within internal tissues. Together, these compounds influence colonization efficiency and the expression of plant growth-promoting traits in associated bacteria [47].

## 3. Plant Growth-Promoting Rhizobacteria Associated with *Coffea arabica and Coffea canephora*

PGPR are beneficial root-associated microorganisms that colonize the rhizosphere, the narrow soil zone influenced by plant roots [48,49]. Over the past decade, studies across coffee-growing regions in Latin America, Asia and Africa have underscored the importance of these plant-microbe interactions in both *C. arabica* and *C. canephora*. Functionally, coffee-associated PGPR solubilize phosphate; fix atmospheric nitrogen via BNF; produce auxins; synthesize siderophores; and express ACC deaminase activity. Indirect benefits include the production of antifungal and nematicidal metabolites, secretion of hydrolytic enzymes and elicitation of ISR. Under greenhouse conditions, inoculation commonly improves germination, shoot and root biomass, nutrient uptake and tolerance to drought or nutrient limitation. Notable biocontrol activity against fungal phytopathogens and plant-parasitic nematodes has also been documented.

Nguyen et al. [50] evaluated bacteria isolated from the rhizosphere of robusta coffee (*C*. *canephora*) for their capacity to suppress plant-parasitic nematodes and to promote host growth. Two hundred isolates were obtained on tryptic soy agar (TSA) and identified by 16S rRNA gene sequencing. Twenty-four displayed strong nematicidal activity, each reducing second-stage juveniles (J2) populations of *Meloidogyne* spp., a major pest of *C. canephora* in Vietnam’s Central Highlands, by more than 60%. Among the isolates, *Pseudomonas aeruginosa* caused 98.2% mortality of second-stage juveniles (J2) and inhibited egg hatching by 84.0%. In greenhouse assays, *P. aeruginosa* reduced nematode densities in the coffee rhizosphere by 83.2% and enhanced plant performance, increasing shoot length to 47.7–48.1 cm and root biomass to 30.2–30.4 g per ten plants. The strain also inhibited *Fusarium solani* mycelial growth by 70.5% and secreted several phytohormones such as IAA (231 µg mL^−1^), gibberellic acid (2702 µg mL^−1^), kinetin (36.8 µg mL^−1^) and zeatin (7.71 µg mL^−1^) (Table 1).

Suharjono and Yuliatin [51] isolated PGPR from *C. canephora* and *C. arabica* cultivated in agroforestry systems in the Malang region of East Java, Indonesia. The soils at the sampling sites were strongly acidic, with pH values of 4.5 for *C. canephora* and 4.0 for *C. arabica.* Colonies obtained by serial dilution plating on TSA were screened on Pikovskaya agar for phosphate solubilization and on nitrogen-free semi-solid bromothymol-blue (Nfb) medium for nitrogen fixation. Cultures were incubated for 48 h (IAA production), 72 h (tricalcium phosphate solubilization) or 5 d (nitrogen fixation). Isolates with the highest activities were identified by 16S rRNA gene sequencing. The best-performing strains for each trait were *Bacillus subtilis* DSM 10, which produced 104.4 µg mL^−1^ IAA and solubilized 2.35 µg mL^−1^ P; *Bacillus wiedmannii*, which fixed 2.07 µg mL^−1^ ammonium; *Bacillus* sp., which produced 88.12 µg mL^−1^ IAA; *Pseudomonas putida*, which solubilized 4.5 µg mL^−1^ P; and *Bacillus methylotrophicus*, which fixed 21.5 µg mL^−1^ ammonium. 

Kunwar et al. [52] isolated phosphate-solubilizing bacteria (PSB) from the rhizosphere of *C. arabica* cultivated in Himalayan organic coffee plantations in Khawa, Kavre, Nepal. Isolation was performed by serial dilution on Pikovskaya’s agar, with plates incubated for five days. Of the 20 bacterial isolates obtained, the 8 most efficient strains (PSB1, PSB2, PSB8, PSB10, PSB11, PSB14, PSB19 and PSB20) were selected based on their tricalcium phosphate solubilization index (TSI). These isolates exhibited strong phosphate-solubilizing activity across different culture media. On Pikovskaya’s agar, PSB10 showed the highest tricalcium phosphate solubilization (407.5 µg mL^−1^). In NBRIP medium, PSB11 reached the highest value (529.1 µg mL^−1^), whereas in NBRIP-RP medium, PSB10 achieved the greatest rock-phosphate solubilization (263.3 µg mL^−1^). Four isolates (PSB1, PSB10, PSB11 and PSB20) were further evaluated for their effects on seed germination and seedling growth of coffee under greenhouse conditions. Isolate PSB20 significantly improved plant performance, increasing shoot length (16.1 cm), fresh shoot biomass (5.63 g) and fresh root biomass (4.32 g), outperforming even the application of single superphosphate fertilizer (containing 16% water-soluble P_2_O_5_, 11% sulfur and 21% calcium). PSB1 and PSB20 increased chlorophyll content in leaves. PSB10 increased protein content in leaves to 0.232 mg g^−1^. The isolates were characterized using standard morphological and biochemical assays, including Gram staining, citrate utilization, indole production and the methyl red–Voges–Proskauer (MR-VP) tests. The resulting biochemical profiles were consistent with assignment to the genus *Pseudomonas* (Table 1). 

Navarro et al. [53] isolated and characterized rhizobacteria associated with *C. canephora* at the National Coffee Research, Development and Extension Center in Cavite, Philippines. The soil at the study site was moderately acidic (pH 5.7). Bacteria were isolated via serial dilution and cultured on Pikovskaya agar incubated for five days. Six isolates capable of solubilizing tricalcium phosphate (PCL 1.2, PCL 2.1, PCL 2.3, PCR 1.1, PCR 1.3 and PCR 1.5) were identified, with solubilization indices ranging from 2.5 to 3.5. Regarding biocontrol traits only PCR 1.3 produced HCN, while hydrolytic enzyme activity varied among isolates: PCL 2.1 produced amylase; PCR 1.1 secreted protease; PCR 1.3 produced both amylase and protease; and PCR 1.5 produced amylase, protease and pectinase. Moreover, the isolates demonstrated tolerance to various abiotic stress conditions. All isolates tolerated a pH range of 4 to 11 and both PCL 1.2 and PCL 2.1 withstood up to 7% NaCl. Tolerance to heavy metals was also assessed: most isolates tolerated 1600 ppm of MnSO_4_, except PCR 1.3, which tolerated only 800 ppm. PCR 1.1 tolerated up to 800 ppm of CuSO_4_ and all isolates tolerated 800 ppm of Pb(C_2_H_3_O_2_)_2_. Based on 16S rRNA gene sequencing, isolate PCR 1.1 was identified as *Burkholderia cepacia*, PCR 1.3 as *Bacillus sanguinis* and PCR 1.5 as *Burkholderia* sp.

Abawari et al. [54] isolated PSB from the rhizosphere of *C. arabica* at the Jimma Agricultural Research Center (Ethiopia). Sampling was conducted at three sites: Seka Cheorsa (shade-grown coffee; soil pH 4.6), Mena (traditional plantations; pH 5.5–5.6) and Goma (cultivated plantations and experimental fields; pH 6.2). Isolates were recovered by serial dilution and plating on Pikovskaya’s agar, followed by incubation for six days. The isolates were identified based on their biochemical characteristics using the identification keys in Bergey’s Manual of Determinative Bacteriology. Of the 154 isolates, 12 were selected for further characterization; the most promising were RScB1.19 and RMaB2.11, both *Bacillus* spp. Their TSIs were 3.56 and 3.09, respectively, with corresponding solubilized phosphate concentrations of 361.46 and 327.32 µg mL^−1^. Both isolates produced IAA and exhibited BNF capacity. HCN production was assessed qualitatively by a color change (yellow to reddish-brown) on sodium-picrate impregnated filter paper, indicating strong HCN production and yielding a positive result for RScB1.19. Both isolates produced ammonia (NH_3_), tolerated heavy metals (Hg, Cu, Mn and Zn) up to 400 µg mL^−1^ and withstood 3–5% (w/v) NaCl. Furthermore, RScB1.19 and RMaB2.11 showed high efficacy in germination assays using *C. arabica* seeds (var. 74110), RScB1.19 achieved a 30% germination rate with a vigor index of 75, while RMaB2.11 recorded a vigor index of 52. In seedling development, RScB1.19 produced shoot and root lengths of 1.33 cm and 1.17 cm, respectively, whereas RMaB2.11 produced shoots of 1.23 cm and roots of 1.00 cm (Table 1).

Kejela et al. [41] isolated *Bacillus* spp. from the rhizosphere of *C. arabica* in Karnataka, India. Bacteria were obtained by serial dilution on Pikovskaya’s agar supplemented with tricalcium phosphate. Among the 42 isolates, strain BT42 showed the strongest antagonistic activity, inhibiting mycelial growth of the phytopathogens *Colletotrichum gloeosporioides* and *Fusarium oxysporum* by 78 and 86%, respectively. Beyond its biocontrol activity, BT42 exhibited several plant growth-promoting traits, including production of IAA (14.56 µg mL^−1^), solubilization of tricalcium phosphate (6.36 µg mL^−1^), siderophore production (75.9 siderophore units) and ACC deaminase activity. Taxonomic identification based on 16S rRNA gene sequencing indicated that BT42 is closely related to *Bacillus amyloliquefaciens*. The isolate also produced hydrolytic enzymes, including chitinases, β-1,3-glucanases, proteases and lipases. In vitro germination trials showed that inoculation with *B. amyloliquefaciens* significantly increased the germination rate of coffee seeds from 50 to 88.8% (Table 1).

Teshome et al. [55] isolated and characterized PSB associated with *C. arabica* in monoculture systems, agroforestry systems and natural forests in the Kaffa and Jima regions of southwestern Ethiopia. Soil pH at the study sites ranged from 5.0 to 6.8. Bacterial isolates were obtained by serial dilution and plating on Pikovskaya’s agar; plates were incubated for seven days. Of the 169 isolates obtained, 55 were selected in vitro based on the formation of tricalcium phosphate solubilization halos, with a TSI ranging from 0.53 to 6.1. Biochemical and phenotypic identification was conducted using the Biolog GEN III MicroPlate™ system, which enabled determination of the predominant microbial taxa in the *C. arabica* rhizosphere. The identified genera included *Pseudomonas*, *Bacillus*, *Rhodococcus*, *Gordonia* and *Citrobacter*. Among the most efficient PSB were *Pseudomonas tolaasii* and *Citrobacter gillenii*.

Cisneros et al. [56] isolated bacteria capable of solubilizing calcium-, aluminum- and iron-phosphates from the rhizosphere of *C. arabica* cv. Caturra in Palmira, Colombia. The soil at the study site was classified as Typic Melanudands with a pH of 5.08. Two bacterial strains were identified: *Kocuria* sp. and *Bacillus subtilis*. To evaluate their effects on P availability in the growth substrate, a greenhouse assay was conducted by inoculating coffee seedlings with bacterial suspensions at 1 × 10^8^ colony-forming units (CFU) mL^−1^. Treatments included T2 (*Kocuria* sp.), T3 (*B. subtilis*) without rock phosphate (RP), T13 (*Kocuria* sp. + RP) and T14 (*B. subtilis* + RP), using a 1:1 (*w*/*w*) mixture of soil and decomposed coffee pulp as the substrate. Seedlings treated with *B. subtilis* (T3 and T14) exhibited higher concentrations of available P in both the substrate and leaf tissues than those inoculated with *Kocuria* sp. (T2 and T13). Foliar P in plants treated with *B. subtilis* was approximately 2300 mg kg^−1^, whereas *Kocuria*-treated plants averaged about 2000 mg kg^−1^. These results indicate a greater potential of *B. subtilis* as a phosphate-solubilizing biofertilizer (Table 1).

Satyaprakash et al. [57] isolated PSB associated with *C. arabica* grown in shade-cultivated plantations within semi-evergreen forests of the Visakhapatnam District, Eastern Ghats, India. Bacteria were obtained using the serial-dilution plate method on Pikovskaya’s agar and incubated for 96 h. During in vitro assays, colonies exhibiting distinct halos indicative of tricalcium phosphate solubilization were selected for subsequent characterization. Fifteen phosphate-solubilizing isolates were recovered and identified based on morphological and biochemical characteristics following the keys in Bergey’s Manual of Systematic Bacteriology. *Bacillus* was the predominant genus, followed by *Pseudomonas*. Species identified among the isolates included *Bacillus cereus*, *Bacillus subtilis*, *Bacillus megaterium*, *Pseudomonas fluorescens*, *Pseudomonas mallei*, *Enterobacter aerogenes*, *Klebsiella aerogenes*, *Proteus vulgaris*, *Proteus mirabilis*, *Staphylococcus aureus*, *Streptococcus* sp., *Corynebacterium kutscheri*, *Corynebacterium xerosis*, *Micrococcus varians* and *Mycobacterium bovis.*

Jasso-Arreola et al. [25] isolated rhizobacteria with ACC deaminase activity from *C. arabica* L. var. Costa Rica 95 cultivated at the INIFAP Experimental Field in Veracruz, Mexico. Isolation was performed on a selective mineral medium in which ACC served as the sole N source, enriching for bacteria capable of catabolizing this compound. Based on 16S rRNA gene sequencing, twelve strains were assigned to seven genera within the classes Gammaproteobacteria and Bacteroidia: RCa01 and RCa28, *Serratia* spp.; RCa07, *Stenotrophomonas* sp.; RCa08 and RCa20, *Sphingobacterium* spp.; RCa12, *Raoultella* sp.; RCa13, *Chryseobacterium* sp.; RCa31, RCa37 and RCa62, *Pantoea* spp.; and RCa18 and RCa58, *Pseudomonas* spp. Plant growth-promoting traits were quantified, including phosphate solubilization, IAA production, ACC deaminase activity, siderophore production and BNF. Tricalcium phosphate solubilization, expressed as the TSI on NBRIP medium, was highest in the *Pantoea* isolates (TSI = 2.3–3.2). These strains also produced elevated IAA levels (up to 30.23 µg mL^−1^) and exhibited notable ACC deaminase activity (9.32 µmol α-ketobutyrate mg^−1^ protein h^−1^). Five of the twelve strains secreted siderophores, with RCa01 forming the widest halo (7.0 mm). Under in vivo conditions, the physiological impact of inoculation on coffee seedlings was assessed under controlled water deficit (55% WHC). Inoculation with RCa62 substantially enhanced root development (total root length = 138.9 m; root dry mass = 10.57 g), relative leaf water content, shoot biomass (14.9 g) and bud formation (4.7 buds; 1.57 g fresh mass) compared with uninoculated controls. RCa62-treated plants also accumulated less proline under drought, consistent with a reduced perception of water stress (Table 1).

Nuguse and Kejela [58] isolated actinobacteria with biocontrol potential from the rhizosphere of *C. arabica* grown under the Yayo agroforestry coffee-forest system in south-western Ethiopia. Eighty-two isolates were recovered on selective media and assigned to genus level using morphological, physiological and biochemical criteria. Antagonism against *Gibberella xylarioides*, the causal agent of coffee wilt disease, was assessed in vitro by the dual-culture method. Four isolates (MUA13, MUA14, MUA26 and MUA52) inhibited mycelial growth by >70%, with MUA26 achieving 83.3% inhibition. These isolates were subsequently applied to coffee seedlings in a greenhouse, where they significantly reduced disease incidence and severity; again, MUA26 was the most effective, providing 83.3% pathogen suppression and lowering wilt severity to 16.7%. Isolate MUA26 also produced the hydrolytic enzymes chitinase, protease and lipase and solubilized tricalcium phosphate and zinc, underscoring its dual role as a biocontrol agent and plant-growth promoter. Phenotypic and biochemical profiles placed all four isolates in the genus *Streptomyces* (Table 1).

de Souza et al. [59] investigated nine PGPB isolates obtained from the rhizosphere of Brazilian coffee plants, *C. arabica* cultivars Catuaí Amarelo and Obata and *C. canephora* cv. Robusta, using serial dilution plating on selective media. Identification based on 16S rRNA gene sequencing classified the isolates into the genera *Pseudomonas*, *Bacillus* and *Enterobacter.* From *C. arabica*, the isolates included Mn2 (*Enterobacter mori*), Mn3a (*Bacillus safensis*), Ob2 (*Pseudomonas gozinkensis*) and Ob3a (*Bacillus safensis*), while from *C. canephora*, the isolates were Rb1 (*Bacillus cereus*), Rb2a (*P. gozinkensis*), Rb2b (*P. koreensis*), Rb3a (*P. koreensis*) and Rb3b (*Pseudomonas* sp.). Functional characterization revealed a high degree of metabolic diversity among the isolates. Rb2a, Mn3a, Ob2, Mn2, Rb3a and Rb3b were capable of synthesizing IAA or related indolic compounds. HCN production was observed exclusively in *C. canephora* isolates, specifically Rb2a, Rb1, Rb3a and Rb3b. Dicalcium phosphate solubilization was detected in Rb1, Rb3a, Rb3b and Ob2. Siderophore production was demonstrated by Rb2a, Rb1, Mn3a, Ob2 and Rb3a. ACC deaminase activity was exhibited by Rb2a, Mn3a, Ob2, Mn2, Ob3a and Rb3b. Catalase activity was present in all strains except Rb2b. Additionally, exopolysaccharides (EPS) production was confirmed in Ob2, Mn2, Rb3a and Rb3b. In greenhouse assays, inoculation with each of nine bacterial isolates in seedlings of *C. arabica* enhanced plant growth, improved nutrient status and suppressed disease, with the magnitude of these effects varying among treatments. Rb2a produced the most pronounced increases in plant height, number of leaves and total biomass, whereas Mn2 and Ob3a improved all measured growth metrics, including shoot and root dry mass. Isolates Rb3b and Rb2b primarily stimulated shoot and root biomass accumulation. Nutrient profiling showed that Rb2a, Mn2, Ob3a and Rb2b raised foliar concentrations of the macro and micronutrients N, P, K, Ca, Mg, Mn, Zn and B; Rb3a elevated P, K, Ca, Mg, S, Mn, Cu, Zn and B; Rb3b increased P, K, Mg, S, Mn, Cu, Zn and B; and Ob2 mainly enhanced Ca, Mg, Mn, Cu and Zn uptake. The isolates also displayed differential biocontrol efficacy. Rb3a suppressed coffee rust caused by *H. vastatrix* by 98.5%, Rb3b achieved a 58% reduction and Ob3a provided 90% control of leaf spot caused by *Boeremia coffeae*. Collectively, these findings highlight the broad functional repertoire of Brazilian coffee-rhizosphere PGPB and underscore their promise for integrated nutrient management and disease mitigation in coffee cultivation (Table 1).

Nguyen et al. [60] isolated IAA-producing bacteria from the rhizosphere of *C. canephora* plants cultivated in Dak Nong Province, Vietnam. Using TSA, they obtained 319 isolates. IAA production was quantified with the Salkowski colorimetric assay. Isolate DN13-B03 produced the highest IAA level (178 µg mL^−1^). Based on 16S rRNA gene sequencing, this isolate was identified as *Priestia megaterium*.

**Table 1:** Rhizobacterial taxa evaluated in functional assays for plant growth promotion and biological control under greenhouse conditions in *Coffea arabica* L. and *Coffea canephora* Pierre ex Froehner worldwide.

Genus/Species	Associated Coffee Species	Plant Growth-Promoting Traits	Effect on the Plant	Country	Reference
*Bacillus subtilis* *Kocuria* sp.	*Coffea arabica* var. caturra	Phosphate solubilization	Phosphorus content in leaves increased Biocontrol activity not assessed	Colombia	[56]
*Bacillus amyloliquefaciens*	*Coffea arabica*	Phosphate solubilization IAA, siderophore and ammonia (NH_3_) production ACC deaminase activity Hydrolytic enzyme secretion (chitinase, β-1,3-glucanase, protease and lipase)	Biocontrol of *Colletotrichum gloeosporioides* and *Fusarium oxysporum* enhances the germination of infected seeds	India	[41]
*Pseudomonas* spp.	*Coffea arabica*	Phosphate solubilization	Germination rate, shoot length, shoot and root biomass increased Chlorophyll and protein content in leaves increased Biocontrol activity not assessed	Nepal	[52]
*Bacillus* sp.	*Coffea arabica*	Phosphate solubilization IAA, NH_3_ and hydrogen cyanide (HCN) production Biological nitrogen fixation capacity Tolerance to heavy metals (Hg, Cu, Zn, Mn) and salinity.	Germination rate, root growth and seedling vigor increased Biocontrol activity not assessed	Ethiopia	[54]
*Pseudomonas aeruginosa*	*Coffea canephora*	IAA, gibberellic acid, kinetin and zeatin production	Shoot length and root biomass increased Chlorophyll a, chlorophyll b and carotenoid concentrations increased Inhibition of *Fusarium solani* F04 and suppression of egg hatching in *Meloidogyne* spp.	Vietnam	[50]
*Streptomyces* sp.	*Coffea arabica*	Phosphate and zinc solubilization Hydrolytic enzyme secretion (chitinase, protease and lipase)	Biocontrol against *Gibberella xylarioides*	Ethiopia	[58]
*Enterobacter mori* *Bacillus* spp. *Pseudomonas gozinkensis*	*Coffea arabica*	IAA-type indolic compound synthesis ACC deaminase activity Siderophore, catalase and exopolysaccharide (EPS) production	Seedling height, number of leaves, shoot biomass, root biomass and total dry biomass increased Biocontrol against *Boeremia coffea*	Brazil	[59]
*Pseudomonas* spp. *Bacillus cereus* *Pseudomonas koreensis* *Pseudomonas* sp.	*Coffea canephora*	IAA-type indolic compound synthesis ACC deaminase activity Siderophore, catalase and EPS production	Seedling height, number of leaves, shoot biomass, root biomass and total dry biomass increased Biocontrol against *Hemileia vastatrix*	Brazil	[59]
*Serratia* spp. *Stenotrophomonas* sp. *Sphingobacterium* spp. *Raoultella* sp. *Chryseobacterium* sp. *Pantoea* spp. *Pseudomonas* spp.	*Coffea arabica* var. Costa Rica 95	ACC deaminase activity Phosphate solubilization IAA and siderophore production Biological nitrogen fixation capacity	Promotion of root development, relative foliar water content, aerial biomass and foliar bud production Biocontrol activity not assessed	Mexico	[25]

ACC, 1-aminocyclopropane-1-carboxylate; IAA, indole-3-acetic acid.

## 4. Endophytic Plant Growth-Promoting Bacteria in *Coffea arabica* and *Coffea canephora*

Endophytic bacteria reside within plant tissues for at least part of their life cycle without causing apparent harm to the host. They colonize multiple organs including roots, stems, leaves and seeds and establish symbiotic associations that confer several benefits to the plant. These benefits include growth promotion via phytohormone production, improved nutrient acquisition and ISR to pathogens. In addition, many endophytes synthesize bioactive compounds with antimicrobial properties [61,62]. Recent studies in *C. arabica* and *C. canephora* show that endophytic bacteria perform multiple plant-beneficial functions, including phosphate solubilization, BNF, production of IAA and HCN, and the secretion of enzymes and metabolites with antagonistic activity against fungal phytopathogens (e.g., *Fusarium*, *Rhizoctonia*) and plant-parasitic nematodes (e.g., *Meloidogyne*, *Pratylenchus*). These endophytes also produce lipopeptides, siderophores and EPS, and exhibit ACC deaminase activity, which collectively modulate root growth, mitigate oxidative stress and enhance tolerance to abiotic stresses.

Asyiah et al. [63] isolated endophytic bacteria from the roots of asymptomatic coffee plants naturally infested with plant-parasitic nematodes at two plantations in East Java, Indonesia: (i) *C. arabica* infested with *Pratylenchus coffeae* and (ii) *C. arabica* infested with *Radopholus similis*. Twenty bacterial isolates were obtained on TSA using a serial-dilution approach and identified by 16S rRNA gene sequencing. In greenhouse bioassays, all isolates significantly reduced nematode penetration into *C. arabica* seedling roots. Isolates SWE, SWF and KBF were the most effective, each reducing penetration by >85%. All three exhibited strong proteolytic activity, suggesting a role for extracellular proteases in nematode suppression. Molecular analyses identified SWE as *Bacillus subtilis*, whereas SWF and KBF were identified as *Bacillus anthracis* (Table 2).

In the study conducted by Hoang et al. [64], endophytic bacteria were isolated from the roots of *C. canephora*. Root segments were cultured on nutrient agar, yeast extract agar and ISP2 medium (International *Streptomyces* Project-2). The inoculated Petri dishes were incubated for periods ranging from 2 to 30 days, yielding a total of 77 endophytic bacterial isolates. Taxonomic identification was carried out through 16S rRNA gene sequencing, allowing classification of the isolates into the genera *Bacillus*, *Serratia*, *Paenibacillus*, *Enterobacter* and *Streptomyces.* Strains of the genus *Streptomyces* exhibited strong antagonistic activity in vitro; consequently, all isolates belonging to this genus were selected for biocontrol assays, particularly against the plant-parasitic nematode *Meloidogyne incognita*, warranting further evaluation as candidate biocontrol agents. In egg-hatching inhibition assays, isolates COG21, CBG31 and COG14 demonstrated varying levels of efficacy. Isolate CBG26 reduced egg hatching by approximately 50%. Notably, CBG9 achieved 85.8% inhibition of egg hatching and 85% mortality in second-stage juveniles (J2), underscoring its high potential as a biocontrol agent.

Pratiwi et al. [65] isolated plant growth-promoting endophytic bacteria from the roots of *C. arabica* and *C. canephora* in coffee plantations located in UB Forest, Malang, East Java, Indonesia, under agroforestry systems. Isolation was performed using the serial dilution technique on selective media TSA for IAA-producing bacteria, Pikovskaya’s agar for phosphate-solubilizing bacteria and a nitrogen-free medium for N-fixing bacteria. Cultures were incubated for 48–72 h. Molecular identification of the isolates was conducted by 16S rRNA gene sequencing. From *C. canephora*, 10 IAA-producing isolates, 8 tricalcium phosphate-solubilizing isolates and 7 N-fixing isolates were obtained. From *C. arabica*, 12 IAA-producing isolates, 7 phosphate-solubilizing isolates and 6 nitrogen-fixing isolates were identified. Among the *C. canephora* isolates, those with the highest apparent potential were SS.E2 (*Bacillus cereus* ATCC 14579), which produced 110.73 µg mL^−1^ IAA; SS.P3 (*Rahnella aquatilis*), which solubilized 4.42 µg mL^−1^ phosphate and SS.N2 (*R. aquatilis*), which fixed 3.15 µg mL^−1^ N. Among the *C. arabica* isolates, the most active strains were SW.E9 (*Bacillus cereus* ATCC 14579), with IAA production of 257.16 µg mL^−1^; SW.P5 (*Kluyvera intermedia*), with phosphate-solubilization capacity of 4.55 µg mL^−1^; and SW.N6 (*Pseudomonas tolaasii* NCPPB 2192), which exhibited N-fixation activity of 1.16 µg mL^−1^.

Duong et al. [42] isolated endophytic bacteria from the roots and seeds of *C. canephora* cultivated in Vietnam by plating samples on TSA and incubating them for 14 days. Of the 140 isolates obtained, 80 were selected for functional and molecular characterization. Taxonomic identification based on 16S rRNA gene sequencing placed the isolates within three bacterial phyla—Actinobacteria, Firmicutes, and Proteobacteria—encompassing 30 distinct genera. Isolates from *C. canephora* roots included *Bacillus*, *Burkholderia*, *Luteibacter*, *Kitasatospora*, *Lechevalieria*, *Streptomyces*, *Kocuria*, *Nocardia*, *Rhizobium*, *Sphingobium*, *Staphylococcus* and *Bradryrhizobium,* whereas seed-derived isolates corresponded to *Bacillus*, *Brachybacterium*, *Curtobacterium*, *Methylobacterium*, *Paracoccus*, *Kocuria*, *Caballeronia*, *Mycobacterium* and *Nakamurella*. With respect to plant growth-promoting traits, 30 isolates solubilized tricalcium phosphate, 25 produced indolic compounds and 23 synthesized siderophores. Regarding enzymatic activities, esterase was the most prevalent (detected in 34 isolates), followed by lipase, gelatinase and chitinase. Only one isolate produced HCN. Based on these results, 50 isolates were selected for biocontrol assays targeting the fungus *Fusarium oxysporum* and the plant-parasitic nematodes *Radopholus duriophilus* and *Pratylenchus coffeae*. Nematicidal bioassays showed that five *Bacillus* strains achieved 100% mortality of both nematode species within 24 h. In antifungal assays, strains belonging to *Burkholderia*, *Bacillus* and *Streptomyces* inhibited the radial mycelial growth of *F. oxysporum* by more than 40%, underscoring their strong in vitro biocontrol potential (Table 2).

Sharan et al. [66] isolated endophytic PGPB from the leaves of *C. arabica* grown at the Regional Coffee Research Station in Thandigudi, India. Leaf samples were plated on yeast–malt extract (YM) agar, yielding seven bacterial isolates. Cultures were incubated for 2–5 days. Molecular identification by 16S rRNA gene sequencing assigned the isolates to *Azospirillum lipoferum*, *Bacillus subtilis*, *Bacillus megaterium*, *Chryseobacterium indologenes*, *Pseudomonas* spp., *Bacillus* spp. and *Pseudomonas putida*. These isolates were subsequently characterized morphologically and biochemically. Among them, *C. indologenes* and *P. putida* were selected for their strong inhibition of phytopathogen growth. In vitro, both strains exhibited antagonistic activity against *Pythium* spp., *Macrophomina* spp. and *Fusarium* spp., reducing radial mycelial growth by 35–72%.

de Castro et al. [67] isolated endophytic bacteria from the leaves of *C. arabica* by culturing macerated leaf fragments on TSA medium, followed by incubation for 48 h. Taxonomic and phylogenetic identification of the bacterial isolates was carried out using the Type Strain Genome Server (TYGS) bioinformatics platform, which allowed classification of the isolates into three bacterial species: *Bacillus mycoides*, *Bacillus thuringiensis* and *Bacillus velezensis*. In vitro assays showed that, among the bacterial isolates evaluated, *Bacillus thuringiensis* displayed the highest efficacy as a biocontrol agent against lepidopteran pests, achieving 100% mortality of *Spodoptera frugiperda* and *Helicoverpa armigera*. Regarding antifungal activity, *B. velezensis* was the most effective isolate, significantly inhibiting the mycelial growth of the plant pathogens *F. oxysporum*, *Rhizoctonia solani* and *Macrophomina phaseolina*, whereas *B. mycoides* showed no relevant activity in fungal suppression. In insect toxicity assays, *B. thuringiensis* maintained strong insecticidal potential, achieving 100% larval mortality in *Diatraea saccharalis*, *S. frugiperda*, *H. armigera* and *Chrysodeixis includens*. *B. velezensis* also exhibited notable insecticidal effects, albeit to a lesser extent, with 83.3% mortality in *S. frugiperda*, 71% in *C. includens* and 58.3% in *H. armigera*. In contrast, *B. mycoides* did not demonstrate significant larvicidal activity. These findings highlight the differential potential of *Bacillus* species as bioinsecticidal and biofungicidal agents, offering promising applications for the integrated management of pests and diseases within the framework of sustainable coffee cultivation.

Ramos-Cabrera et al. [68] isolated inorganic phosphate-solubilizing endophytic bacteria from the roots of *C. arabica* var. Bourbon grown in Andisols on the Popayán Plateau, Colombia. Isolation was carried out by serial dilution and plating on National Botanical Research Institute Phosphate (NBRIP) medium supplemented with tricalcium phosphate; plates were incubated for 72 h. Colonies forming clear halos indicative of phosphate solubilization were selected and the TSI was determined. Of the 18 isolates obtained, End-F-5 and End-F-34 exhibited the highest TSI values (1.6 and 1.7, respectively). Both isolates also formed biofilms and displayed proteolytic activity. Taxonomic analysis based on 16S rRNA gene sequencing indicated that the isolates were assigned primarily to Gammaproteobacteria (72%), Firmicutes (22.3%) and Betaproteobacteria (5.5%). In vitro assays showed that inoculation of coffee seedlings with the isolate End-F-5 (*Staphylococcus succinus*) increased the number of leaves by 4.8%, root dry mass by 20.2%, shoot biomass by 12.6%, and P concentration in leaves by 14%. Co-inoculation with End-F-5 and End-F-34 (*Leclercia adecarboxylata*) also significantly increased the number of leaves (3.7%), stem length (12.3%) and shoot weight (15%) (Table 2). When applied alone, End-F-34 increased stem length by 14.8% and P concentration in leaves by 3.4%.

de Souza et al. [59] isolated endophytic bacteria from the roots of *C. arabica*. The isolates were identified as *Enterobacter hormaechei* (EOb1) and *P. putida* (EMn2a). The plant growth-promoting capabilities of both strains were evaluated, including the biosynthesis of indolic compounds (IAA-type), siderophores, ACC deaminase, catalase, EPS, HCN and dicalcium phosphate solubilization. *E. hormaechei* produced IAA, HCN, siderophores, ACC deaminase, catalase and EPS. *P. putida* produced EPS, IAA, siderophores, ACC deaminase and catalase. Under greenhouse conditions, inoculation with *E. hormaechei* and *P. putida* enhanced plant growth and nutrient uptake in coffee plants. However, *E. hormaechei* produced a more pronounced effect, significantly increasing plant height, number of leaves, shoot dry biomass, root dry biomass and total dry biomass, as well as the foliar content of macro- and micronutrients (N, P, K, Ca, Mg, S, Fe, Mn, Cu, Zn, and B). Additionally, *E. hormaechei* exhibited biocontrol activity, reducing *H. vastatrix* urediniospore germination by 91% and inhibiting the mycelial growth of *Boeremia coffeae* by 50% (Table 2).

Ogata-Gutiérrez et al. [69] isolated endophytic bacteria from leaves of *C. arabica* cultivated in Chanchamayo, Peru. Surface-sterilized leaf segments were plated on Yeast Extract Mannitol (YEM) agar and incubated for 15 days. The endophytic bacterial density was estimated at 2.1 × 10^6^ CFU per gram of tissue. Isolate CSEDT7 exhibited in vitro antifungal activity against *Mycena citricolor*, *Colletotrichum* sp. and *H. vastatrix*. Analysis of the 16S rRNA gene identified this isolate as *Luteibacter anthropi*, with 99.6% sequence identity to reference sequences in GenBank.

**Table 2:** Endophytic taxa evaluated in functional assays for plant growth promotion and biological control under greenhouse conditions in *Coffea arabica* L. and *Coffea canephora* Pierre ex Froehner worldwide.

Genus/Species	Associated Coffee Species	Plant Organ of Isolation	Plant Growth-Promoting Traits	Effect on the Plant	**Country**	**Reference**
*Bacillus subtilis* *Bacillus anthracis*	*Coffea arabica*	Root	Proteolytic activity	Biocontrol against *Pratylenchus coffeae*	Indonesia	[63]
*Bacillus* spp. *Burkholderia* spp. *Luteibacter* sp. *Kitasatospora* sp. *Lechevalieria* sp. *Streptomyces* sp.	*Coffea canephora*	Root	Phosphate solubilization Indolic compounds (IAA-type) synthesis Siderophore, HCN, esterase, lipase, gelatinase and chitinase production	Biocontrol against *Fusarium oxysporum*, *Radopholus duriophilus* and *Pratylenchus coffeae*	Vietnam	[42]
*Bacillus* spp. *Curtobacterium* spp. *Brachybacterium* sp. *Methylobacterium* sp., *Paracoccus* sp.	*Coffea canephora*	Seed	Phosphate solubilization Indolic compounds (IAA-type) synthesis Siderophore, HCN, esterase, lipase, gelatinase and chitinase production	Biocontrol against *Fusarium oxysporum*, *Radopholus duriophilus* and *Pratylenchus coffeae*	Vietnam	[42]
*Enterobacter hormaechei* *Pseudomonas putida*	*Coffea arabica*	Root	Phosphate solubilization Indolic compounds (IAA-type) synthesis Siderophore, HCN, esterase, catalase and exopolysaccharides production ACC deaminase activity	Plant height, number of leaves, shoot dry biomass, root dry biomass and total dry biomass increased Biocontrol against *Hemileia vastatrix* and *Boeremia coffeae*	Brazil	[59]
*Staphylococcus succinus* *Lecrercia adecarboxylata*	*Coffea arabica*	Root	Biofilm formation and proteolytic activity	Number of leaves, root dry mass, shoot biomass, stem length, shoot weight and phosphorus content in leaves increased	Colombia	[68]

IAA, indole-3-acetic acid; HCN, hydrogen cyanide; ACC, 1-aminocyclopropane-1-carboxylate.

**Figure 1:**
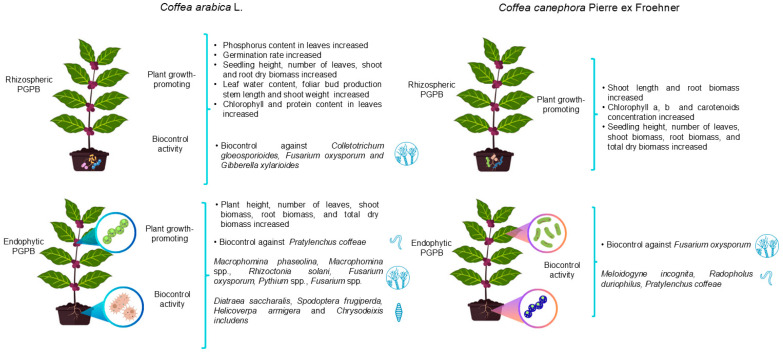
Beneficial effects of rhizospheric and endophytic plant growth-promoting bacteria (PGPB) associated with *Coffea arabica* L. and *Coffea canephora* Pierre ex Froehner on coffee plants under greenhouse conditions.

PGPB associated with *C. arabica* and *C. canephora* offer a sustainable and environmentally friendly alternative to mitigate the negative impacts of excessive agrochemical use in coffee production. Under greenhouse conditions, PGPB have consistently increased shoot and root biomass, raised seed germination rates and enhanced overall plant vigor in coffee plants (Figure 1). In addition, several strains exhibit strong biocontrol activity and can elicit ISR in coffee.

## 5. Possible Roles of Host Filtering, Management and Edaphic Constraints in Shaping Coffea Bacterial Consortia

**Figure 2:**
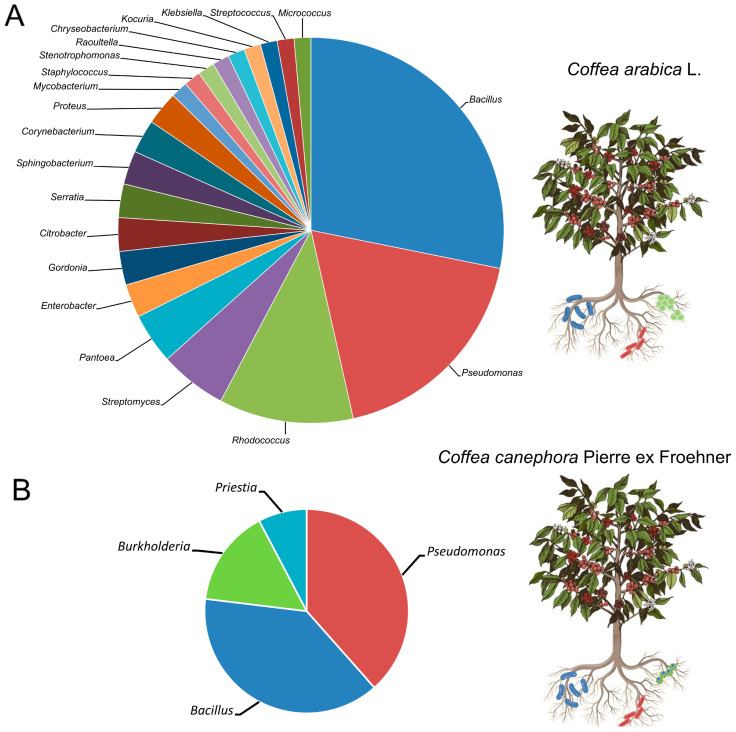
Rhizosphere-associated bacterial genera isolated from (**A**) *Coffea arabica* L. and (**B**) *Coffea canephora* Pierre ex Froehner, ordered by their frequency of occurrence in the literature surveyed. Frequency was computed at the isolate level as the sum of isolates per genus reported across studies. Because isolation effort and culture conditions vary among studies, these frequencies should be interpreted as counts of reported isolates rather than standardized relative abundances.

**Figure 3:**
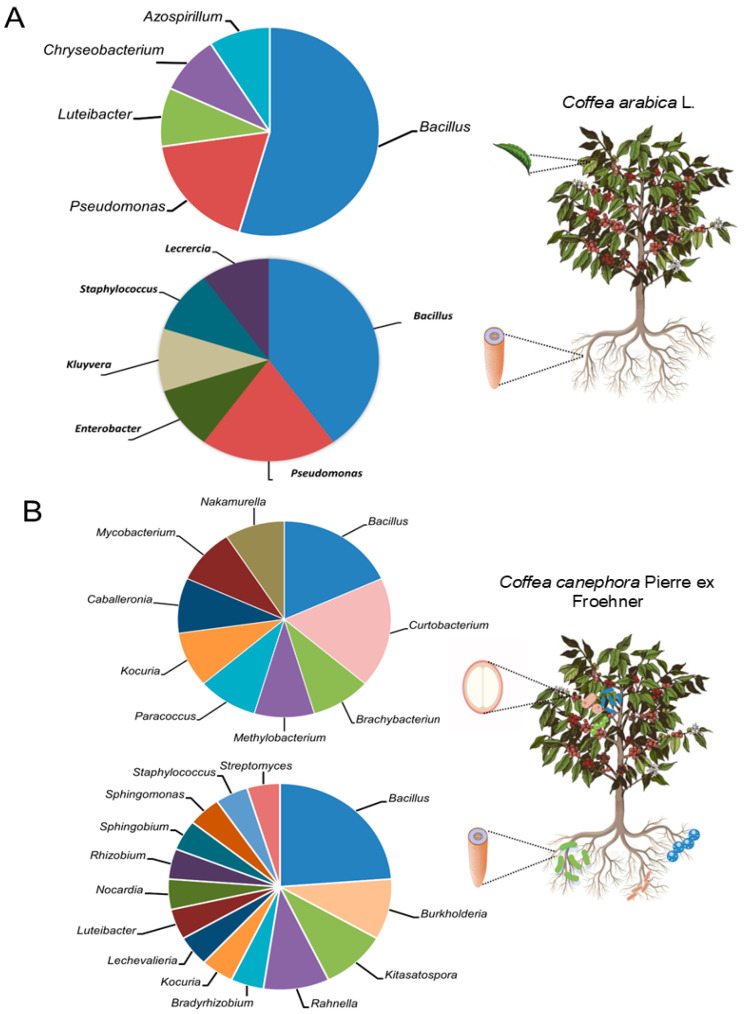
Endophytic bacterial genera isolated from (**A**) roots and leaves of *Coffea arabica* L. and (**B**) roots and seeds of *Coffea canephora* Pierre ex Froehner, ordered by their frequency of occurrence in the surveyed literature. Frequency was computed at the isolate level as the sum of isolates per genus reported across studies for the indicated compartment. Because isolation effort and culture conditions vary among studies, these frequencies should be interpreted as counts of reported isolates rather than standardized relative abundances.

A compartment-specific core microbiome is evident. In the rhizosphere of both hosts (*C. arabica* and *C. canephora*), *Bacillus* and *Pseudomonas* consistently dominate (Figure 2A,B). Within endophytic communities, *Bacillus* is the dominant genus across plant tissues, including roots, leaves and seeds (Figure 3), whereas accompanying genera are host- and tissue-specific. In *C. arabica*, endophytes frequently include *Pseudomonas* in roots and leaves (Figure 3A). In *C. canephora*, root endophytes recurrently include *Burkholderia*, *Kitasatospora* and *Rahnella* (Figure 3B), while seed endophytes are enriched for *Curtobacterium* (Figure 3B). These divergences are consistent with host-specific filters, potentially arising from root exudate profiles, root anatomy and microbial transmission pathways. Likewise, management practices that modulate stress (e.g., monoculture, agroforestry, organic management) tend to restructure the consortia. Water deficit favors functions such as ACC deaminase activity and EPS production, whereas pest pressure is associated with enrichment of lipopeptide, siderophore and hydrolytic enzyme producers. Antagonism patterns also differ: in *C. arabica*, responses mediated by *Bacillus* are common (antimycotic lipopeptides, siderophores and activation of ISR). By contrast, *C. canephora* more frequently exhibits mechanisms targeting nematodes and fungi that combine cell wall-degrading enzymes, volatile organic compounds and nutrient competition with documented contributions from *Bacillus* (nematicidal activity), *Burkholderia* (fungal inhibition) and *Pseudomonas* (nematicidal and antifungal effects alongside phytohormone production). Finally, acidic soils with limited P availability promote the selection of phosphate-solubilizing and siderophore-producing taxa. Collectively, differences in dominant genera between *C. arabica* and *C. canephora* likely emerge from the interaction of host filters with biotic and abiotic pressures modulated by management, local microclimates and edaphic constraints.

## 6. Practical Implementation Challenges of Plant Growth-Promoting Bacteria in Coffee Cultivation

Adoption of PGPB in coffee systems poses challenges that extend beyond the laboratory. In nurseries and in the field, inoculation routes ranging from seed treatments and seedling dips to root coating at transplanting, fertigation or foliar spraying for endophytes determine whether bacteria reach the appropriate niches (rhizoplane, endosphere, phyllosphere) and establish persistent colonization. Recent reviews of the coffee microbiome underscore the importance of precise niche targeting and inoculation strategy [70]. After application, microbial persistence may be limited by UV radiation, desiccation, temperature fluctuations, soil pH and agrochemical residues, as well as competition with the native microbiota. Consequently, compatibility with fungicides should be verified explicitly within integrated management programs [71].

Formulation is equally decisive. The choice of carrier (e.g., alginate, biochar) and the use of protectants (e.g., trehalose) govern viability, shelf life and release in the rhizosphere. In this regard, microencapsulation has shown clear improvements in the stability and field performance of PGPB [72]. From an agronomic standpoint, synchronizing inoculation with irrigation, shading and mineral nutrition is critical to ensure that functions such as phosphate solubilization, siderophore production, ACC deaminase activity and ISR are expressed where and when the crop requires them [70].

Compared with single-strain products, multi-strain consortia typically achieve greater efficacy by integrating complementary traits such as nutrient mobilization, tolerance to environmental stressors and pathogen suppression that enhance the robustness of plant responses. However, the magnitude of this benefit depends on rational strain selection, compatibility among strains and appropriate dosing to avoid antagonism or imbalance within the consortium. The superiority of consortia over individual strains is supported by a recent meta-analysis conducted in non-sterile soils across diverse cultivation conditions [73].

Finally, compliance with biosafety requirements and the prevailing regulatory framework is essential. It is necessary to demonstrate the absence of risk determinants such as virulence factors and transferable antimicrobial resistance genes while providing clear safe use guidelines for personnel and establishing mechanisms for environmental traceability. In the European Union, microbial biostimulants are governed by Regulation (EU) 2019/1009 on fertilizing products and by guidance documents that specify safety, labeling and product assessment criteria based on published data [74].

## 7. Conclusions and Perspectives

This synthesis reveals a clear, compartment-specific core microbiome in coffee. In the rhizosphere of both hosts (*C. arabica* and *C. canephora*) *Bacillus* and *Pseudomonas* consistently dominate. Within endophytic communities, *Bacillus* predominates across tissues (roots, leaves and seeds), whereas accompanying genera are host- and tissue-specific. In *C. arabica*, endophytes frequently include *Pseudomonas* in roots and leaves. In *C. canephora*, root endophytes recurrently include *Burkholderia*, *Kitasatospora* and *Rahnella*, whereas seed endophytes are enriched for *Curtobacterium*. Collectively, these patterns indicate strong host- and tissue-level filtering and point to distinct ecological assembly processes, with direct implications for targeted microbiome management in coffee agroecosystems.

PGPB associated with coffee offer a viable pathway for sustainable intensification. To translate these insights into reliable, scalable products, we recommend four integrated priorities: (1) apply strain resolved multiomics to link host specific taxa to functions clearly expressed within the plant; (2) develop bioformulations suitable for on farm deployment, aligned with agronomic management and supported by stability and shelf life analyses; (3) conduct multi-site, multi-season field trials that compare rationally designed consortia with single strains and quantify colonization, persistence, yield and quality gains, nutrient use efficiency and economic returns; and (4) strengthen biosafety and regulatory assessments based on genomic and phenotypic evidence to support safety determinations, define clear use protocols and implement environmental traceability.

## Data Availability

No new data were created or analyzed in this study. Data sharing is not applicable to this article.
